# Altered Metabolic Signature in Pre-Diabetic NOD Mice

**DOI:** 10.1371/journal.pone.0035445

**Published:** 2012-04-13

**Authors:** Rasmus Madsen, Viqar Showkat Banday, Thomas Moritz, Johan Trygg, Kristina Lejon

**Affiliations:** 1 Computational Life Science Cluster (CLiC), Department of Chemistry, Umeå University, Umeå, Sweden; 2 Division of Immunology, Department of Clinical Microbiology, Umeå University, Umeå, Sweden; 3 Umeå Plant Science Center, Department of Forest Genetics and Plant Physiology, Swedish University of Agriculture Sciences, Umeå, Sweden; University of Bremen, Germany

## Abstract

Altered metabolism proceeding seroconversion in children progressing to Type 1 diabetes has previously been demonstrated. We tested the hypothesis that non-obese diabetic (NOD) mice show a similarly altered metabolic profile compared to C57BL/6 mice. Blood samples from NOD and C57BL/6 female mice was collected at 0, 1, 2, 3, 4, 5, 6, 7, 9, 11, 13 and 15 weeks and the metabolite content was analyzed using GC-MS. Based on the data of 89 identified metabolites OPLS-DA analysis was employed to determine the most discriminative metabolites. In silico analysis of potential involved metabolic enzymes was performed using the dbSNP data base. Already at 0 weeks NOD mice displayed a unique metabolic signature compared to C57BL/6. A shift in the metabolism was observed for both strains the first weeks of life, a pattern that stabilized after 5 weeks of age. Multivariate analysis revealed the most discriminative metabolites, which included inosine and glutamic acid. In silico analysis of the genes in the involved metabolic pathways revealed several SNPs in either regulatory or coding regions, some in previously defined *insulin dependent diabetes (Idd)* regions. Our result shows that NOD mice display an altered metabolic profile that is partly resembling the previously observation made in children progressing to Type 1 diabetes. The level of glutamic acid was one of the most discriminative metabolites in addition to several metabolites in the TCA cycle and nucleic acid components. The in silico analysis indicated that the genes responsible for this reside within previously defined *Idd* regions.

## Introduction

Type 1 diabetes is an autoimmune disease which in contrast to the majority of other autoimmune diseases arises already at young age [1]. The genetic contribution to the disease has been estimated to be ∼50%, including >50 involved loci [2], (www.t1dbase.org). In addition, environmental factors such as infections, diet and climate has been attributed as potential underlying contributors. Although the pathogenesis process in diabetes is so far only detectable as either the appearance of islet-specific autoantibodies and/or the mononuclear infiltration in the islets of Langerhan's, it is evident that this process is preceded by other molecular and cellular events, which in turn could be reflected by the metabolome. In line with this it has been demonstrated that children with genetic predisposition for T1D development and that progress to T1D display an altered serum metabolic profile compared to the group of non-progressors [3], [4]. Specifically the levels of succinic acid and phosphatidylcholin were reduced at birth, and elevated levels of glutamic acid preceded seroconversion, i.e. appearance of autoantibodies specific for insulin and glutamic acid decarboxylase (GAD).

The metabolic changes in relation to time of diabetes onset have also been studied in the congenic BB rat model [5]. An alteration in the metabolic profile was observed in DP rats compared to DR1 and DR2 rats 1–2 weeks prior to diabetes onset, and at this time point, based levels of 17 metabolites, it was possible to discriminate progressors versus non-progressors. In line with the studies in humans, phospholipids and the amino acid isoleucine was altered. However, in the rat model it was not possible to score any metabolic differences at earlier time points than 1–2 weeks prior to disease onset.

The NOD mouse is another vastly used model for T1D [6]. In this strain diabetes develops spontaneously and the pathological process resembles in many ways T1D in humans. Mononuclear infiltration in the Langerhans' islets is detected already from 4–5 weeks whereas overt diabetes develops at 3–4 months of age and onwards. T1D in NOD mice is a multigenetic disease where some of the >30 involved loci *(Idds)* [7] are in direct syntenic relationship with some of the human *IDDMs*. Moreover, environmental factors also play an important role where infections, diet and climate clearly has been demonstrated to play a pivotal role [1]. Involvement of different immune cells subsets has been extensively explored and although the disease is transferable with T-cells solely [7], B-cells are without any doubt crucial in the initiation of the pathogenesis process [8]. It is, however, not clear how the disease in NOD mice is initiated.

Recently it was demonstrated that female NOD mice that progress to T1D display an discrete metabolic profile compared to genetically identical individuals that do not develop T1D [9] and this could potentially be explained by differential microbiota in the two groups. However, in this study metabolic alteration due to genetic influences was not tested. In the current study we tested the hypothesis that NOD mice display an altered metabolic signature compared to the non-diabetic C57BL/6 reference strain. In addition we hypothesized that a potential deviation in the metabolism, as if being a contributor to the initiation of the pathogenic process, should be apparent already in young mice, prior to the onset of insulitis.

## Materials and Methods

### Ethics statement, animals and blood sampling

NOD and C57BL/6 mice were originally obtained from Bomholtgaard, Denmark and bred and maintained in the transgenic animal facility (UTCF) at Umeå University. Diabetes incidence in our colony reached 50% in females after 5 months. None of the mice in the study developed diabetes during the sampling period. Experimental procedures were performed in compliance with the relevant Swedish and Institutional laws and guidelines and approved by the Umeå research animal ethic committee (A14-09). Blood samples were taken from individual female mice at the time points 0 weeks (new born) through 6 weeks (n = 8 in all groups except for NOD 3 w n = 7, B6 newborn, 1 w and 5 w n = 7 respectively and B6 6 w n = 5). In order to obtain sufficient blood for analysis at these early time points the animals had to be sacrificed subsequently. From the 7–15 weeks old mice individuals were consecutively sampled (n = 8 in all groups). None of the mice became diabetic throughout the study. After sampling the blood was kept in room temperature for one hour and stored at 4°C over night. Clots was removed, remaining sera spun in a microfuge at 13 000 rpm for five minutes, where after the sera was stored in −80°C until the GC/MS analysis.

### Metabolite extraction and GC-MS analysis

Extraction of metabolites from plasma was undertaken following the protocol of A et al. This method has been shown to produce semi-quantitative data of good linearity, making it appropriate for comparisons across specie as well as time in this study [10]. A detailed flow chart and protocol is provided as Text S1 and Figure S1. In brief, an aliquot, 25 µL, of thawed serum was added to Sarstedt safety cap tubes and 225 µL of the extraction mixture consisting of methanol∶water (9∶1) containing 11 isotopically labeled internal standards were added. The mixture was shaken for 2 min at 30 Hz and stored in an ice-bath for 2 h before centrifugation for 10 min at 4°C and14000 rpm. 200 µL of the supernatant were evaporated to in GC-MS-vials.

The samples were derivatized using methoxyamine and MSTFA as described by A et al. and GC-MS analyses were carried out according to the same protocol.

### Data Processing GC-MS

All non-processed MS-files from the metabolic analysis were exported from the ChromaTOF software in NetCDF format to R (version 2.10.1) in which all data pre-treatment procedures, such as base-line correction chromatogram alignment, data compression and Hierarchical Multivariate Curve Resolution (H-MCR) were performed using custom scripts as described by Jonsson et al. [11]. All manual integrations were performed using in-house R scripts. The data processing protocols resulted in peak areas for the derivatized metabolites and corresponding mass spectra.

### Metabolite libraries, metabolite identification and quantification

The metabolites were identified by comparison of retention indices and mass spectra with data in commercial, as well as in-house, retention indexes and mass spectra libraries using NIST MS Search 2.0 (National Institute of Standards and Technology, 2001). The data processing of the GC-MS data using the H-MCR script resulted in initial datasets. All variables were checked manually and variables originating from internal standards and analytical or processing artifacts excluded.

The dataset was normalized using the 11 added internal standards; a non-centered principal component analysis (PCA) model was built on the basis of the intensity of selected ions originating from the internal standard compounds and all metabolite intensities was divided by the first component from this model [12].

### Statistical analysis

In order to identify trends in the metabolite data metabolite concentration fold changes were calculated for NOD compared to B6 at each time point. This was done by dividing the average concentration in the NOD group with the average concentration in the B6 group. In cases where the fold change was below 1 it was replaced with the negative inverse value for clarity. To obtain a clear overview of the data fold changes was plotted for all time points where the difference was found statistically significant according to two-sided Student's t-tests for samples with equal variance (p = 0.05).

The combination of metabolites that provided the optimal discrimination between NOD and B6 mice was found using Orthogonal Projections to Latent Structures-Discriminant Analysis (OPLS-DA) on unit variance scaled data [13]–[15]. OPLS-DA is a multivariate classification technique that is used for predicting groupings for observations and for characterizing the groups. B6 was set the value zero and NOD the value one, using a dummy matrix. By using 7-fold cross-validation [16] and optimizing the prediction results for models discriminating the two genotypes using backwards variable selection [17] at the time points 0, 3, 4, 5 and 15 weeks the optimal combination of metabolites for describing the differences between NOD and B6. These time points were judged as the most crucial based on the previous analyses.

### Analysis of genetic differences between B6 and NOD in relation to metabolic data

The pathways involved in the metabolism of selected metabolites were collected from the Kyoto Encyclopedia of Genes and Genomes or KEGGs pathway database for mice (http://www.genome.jp/kegg). The metabolic pathways were selected based on metabolomics data and are the pathways where the largest metabolic differences between NOD and B6 were observed. The extent of the pathways was defined by the availability of metabolomics data, i.e. pathways were extended as far as possible with the available data. All enzymes present in a metabolic pathway were examined. Occurrence of the enzymes in *Idd* regions was analyzed using the web resource from www.T1Dbase.org. SNPs in these enzymes were collected from the Mouse Phenome Database using the mouse SNP wizard (http://phenome.jax.org/db/q?rtn=snps/wiz1) listing polymorphisms between the NOD/ShiLtJ and the control C57BL/6J strains. Any SNPs present in the 5′ upstream or 3′ downstream regions were also collected by including 2000 bps on either ends in the search criteria. Non-synonymous SNPs were analyzed for their effect on change of sequence and function using SIFT at http://sift.jcvi.org/. All the analysis was carried out based on build-37 of Entrez Genome View, which is being updated as new SNPs are identified.

## Results

### NOD and B6 mice display discrete metabolic signatures

**Figure 1:**
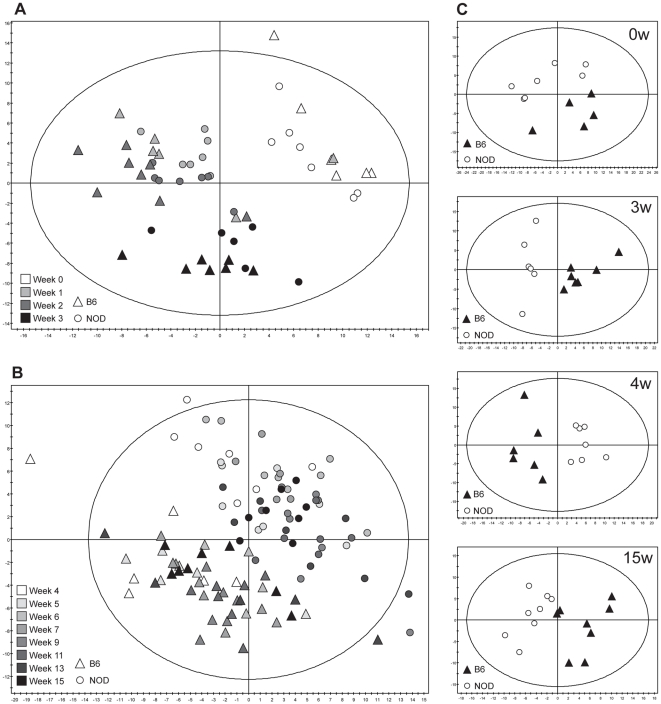
Score plots from Principal Component Analysis (PCA) showing the first (horizontal) and second (vertical) component for all models. A. 0–3 weeks; both NOD and B6 change over time and likewise display divergent metabolic profiles at each time point. The plot accounts for 37.5% of the variation in the metabolite data. B. 4–15 weeks; the metabolic pattern has stabilized and the two strains are readily separable. The plot accounts for 32.5% of the variation in the metabolite data. C. PCA at the chosen time points of 0 w, 3 w, 4 w, and 15 weeks respectively; at each time points the strains are clearly different.

Individual serum samples from NOD and B6 mice (0–15 weeks, n = 184) were analyzed by GC-MS. Based on the levels of 89 identified metabolites an initial overview of the data was obtained by employing Principal Component Analysis (PCA). PCA is an unsupervised statistical technique that summarizes multivariate data in a smaller number of latent variables [18]. This allows identification and visualization of groupings, trends and outliers in the data. A metabolic shift in both strains was observed during the first 0–3 weeks (Figure 1A), a pattern that was stabilized after 4 weeks (Figure 1B). Notably, comparing NOD and B6 mice, the two strains could clearly be distinguished already at 0 weeks (Figure 1C), as well at later time points (Figure 1D–F).

**Figure 2:**
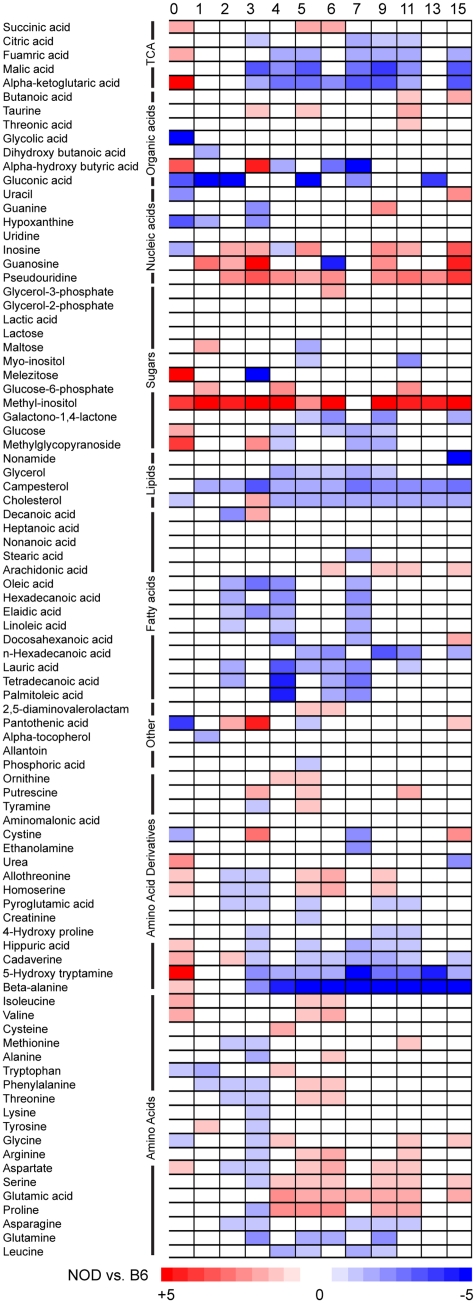
Heat map showing metabolites (that is significantly (p<0.05) different (if labeled in color) in NOD compared to B6 mice. Compounds within the defined classes display similar pattern. The plot is sorted according to compound classes.

To obtain an overview of the individual metabolites that were significantly different between the two strains we calculated fold changes for all metabolites at all time points (Figure 2). Blue and red color indicates that significantly higher respectively lower concentrations were found in NOD compared to B6 mice. The metabolites in the plot were sorted according to compound classes in order to depict general trends in the dataset. We observed that similar compounds showed similar behavior over time, i.e. natural amino acids were generally lower in NOD compared to B6, until four weeks where a general shift to an increased level was found. This shift was caused by a general increase in of amino acids in the week 4 NOD mice compared to week 3. In B6 amino acid levels were generally constant or slightly decreasing from week 3 to 4 (Table S1).

At birth the NOD mice had higher levels of four out of five TCA cycle intermediates and this was reversed at week four where four of these compounds (citric acid, fumaric acid, malic acid and alpha-ketoglutaric acid) showed a large increase in B6 but not in NOD (see Text S1, Table S1, Figures S1 and S2). These new levels were maintained until 15 weeks of age with four of these compounds showed a significant decrease in NOD relative to B6. In addition from four weeks of age NOD had lower levels of most measured free fatty acids; the notable exception being arachidonic acid which from week seven was increased. We also observed that methyl-inositol and pseudouridine differentiated the stains at all ages by being consistently increased in NOD compared to B6.

### The most discriminative metabolites include inosine and glutamic acid

**Figure 3:**
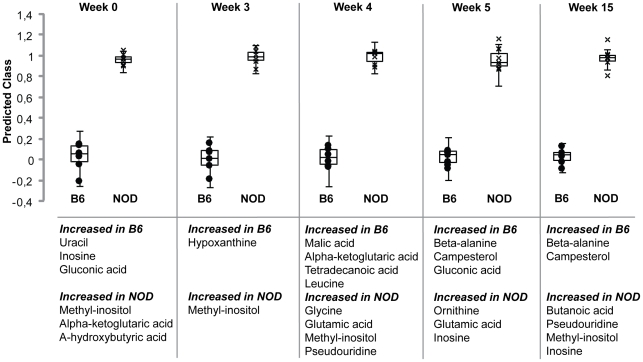
Backward variable selection was used to find the metabolites that were most discriminative between the stains. Box-Whisker-plots show the prediction of stain by the OPLS model with the best predictive ability at five different time points. Values close to zero indicate that the individual was predicted as B6 and values close to one that it was predicted as NOD. The metabolites included in the respective models are designated below.

As the PCA analysis and statistical tests of metabolite differences indicated major changes in the metabolic profiles the first weeks of age (i.e. weeks 3–5) we performed a more thorough analysis for these time points, also including the 0 and 15 weeks of age time points as reference time points. To identify the metabolites discriminating the two stains the most at the five time points, multivariate models with reduced number of metabolites was tested until separation of the mouse strains started deteriorating. The model that provided the optimal separation between the two stains was determined, and the including metabolites in this model is presented in Figure 3 and Figure S2. Metabolites belonging to the nucleic acid group (i.e. uracil, pseudouridin, inosine and hypoxanthine) as well as to the TCA cycle and related amino acids (i.e. alpha-ketoglutaric acid and glutamic acid) were among the most disciminative metabolites.

### Pathway analysis reveals potential responsible enzymes and targeted receptors

**Figure 4:**
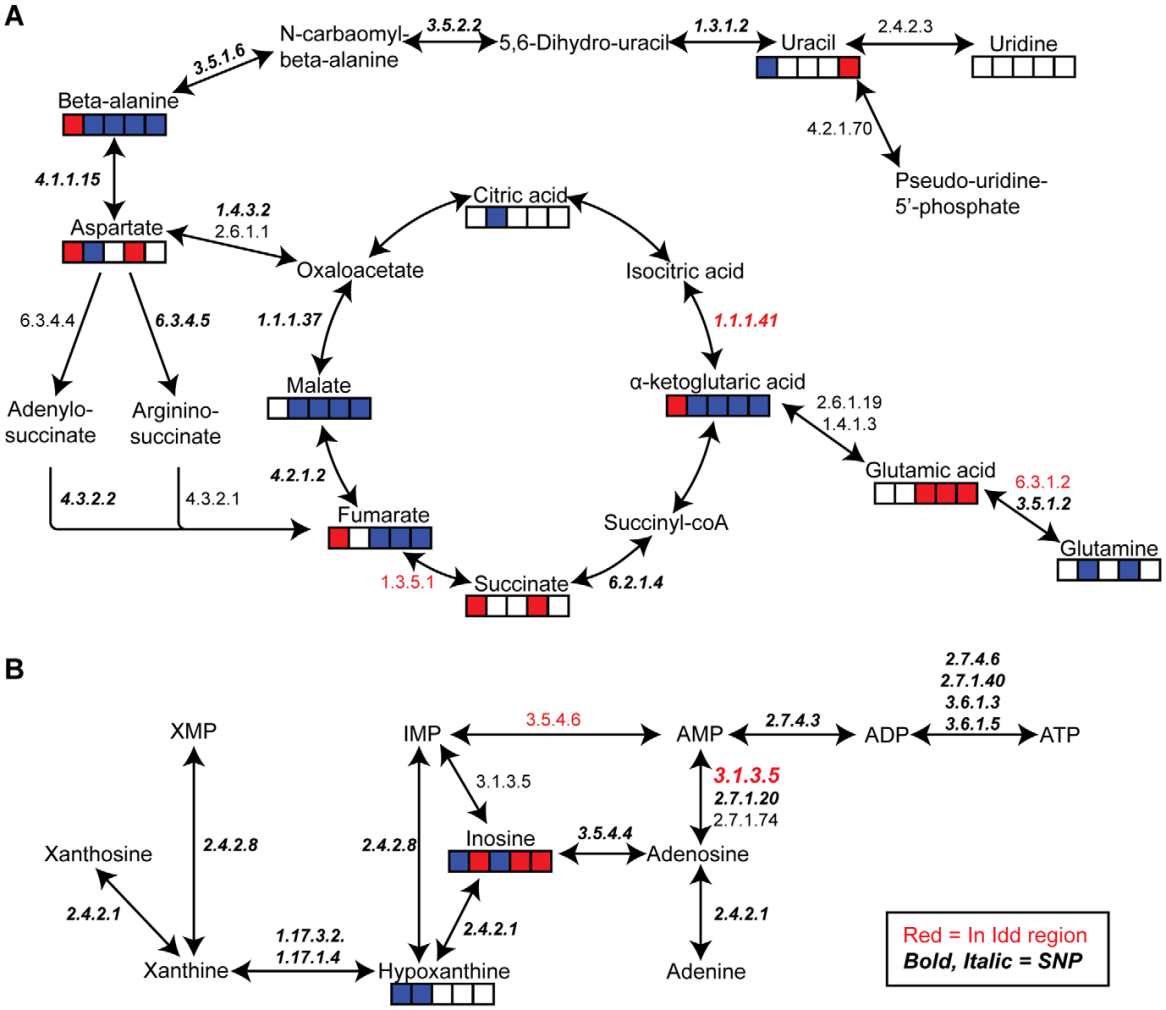
Metabolic pathways possibly responsible for the differences observed in NOD and B6 mice. Metabolite concentrations at weeks 0, 3, 4, 5 and 15 are indicated with colors. Red indicates significantly increased in NOD, blue indicates significantly increased in B6 and white indicates no significant difference. Enzymes involved in metabolism are also show in the plot. Red text indicates that the gene is in *Idd* region and bold, italic text indicates SNPs differentiating NOD and B6 mice. A. Metabolism around the TCA cycle with glutamic acid and glutamine. B. Metabolism of nucleic acid compounds.

The observed metabolic differences between NOD and B6 were likely due to alterations in the enzymes participating in the metabolism. Therefore, we analyzed, by using the KEGG data base, which metabolic enzymes and pathways that could be involved in metabolism of the most discriminative metabolites presented in Figure 3. As seen in Figure 4 the most characteristic differences in metabolism between B6 and NOD could be traced to two main pathways; metabolism around the TCA cycle with glutamic acid and glutamine (Figure 4A), and metabolism of nucleic acid compounds (Figure 4A and B). Based on this analysis we selected 32 enzymes and three receptors involved in the two pathways. The corresponding genes for these proteins were analyzed for any potential genetic differences between NOD and B6. The positioning and sequences of the corresponding genes in NOD and B6 were retrieved from the dbSNP database [19]. Our search yielded 1 274 SNPs out of which 9 SNPs were present in exons, 71 in the 5′, 3′ UTRs and the remaining 1 194 in the intron regions (Table 1).

**Table 1:** Genetic variation in the enzymes involved in metabolic pathways of interest.

Name	Gene/E.C Number	Idd	5′ UTR SNP	Non Synonymous Exon SNP(Dangerous/Tolerated)	Intron SNP	3′ UTR SNP
Glutamine synthetase	*Glul/6.3.1.2*	*Idd5.4b/Idd5.4*				
Glutaminase	*Gls/3.5.1.2*				2	
Glutamate dehydrogenase	*Glud1/1.4.1.3*					
4-aminobutyrate aminotransferase	*Abat/2.6.1.19*					
Glutamate oxaloacetate transaminase 1	*Got1,2/2.6.1.1*		1		41	
L-amino acid oxidase 1	*Lao1/1.4.3.2*		2	1 (0/1)	2	
Glutamic acid decarboxylase 1	*Gad1/4.1.1.15*		1		25	
Argininosuccinate synthetase 1	*Ass1/6.3.4.5*				5	
Adenylosuccinate synthase like 1	*Adssl1/6.3.4.4*					
Adenylosuccinate lyase	*Adsl/4.3.2.2*					
Argininosuccinate lyase	*Asl/4.3.2.1*					
Ureidopropionase	*Upb1/3.5.1.6*			1 (0/1)	42	
Dihydropyrimidinase	*Dpys/3.5.2.2*		4		147	3
Dihydropyrimidine dehydrogenase	*Dpyd/1.3.1.2*				33	
Uridine phosphorylase	*Upp1/2.4.2.3*					
Hypoxyxanthine guanine phosphoribosyl transferase	*Hprt/2.4.2.8*				1	
Adenosine monphosphate deaminase 1/2/3	*Ampd1,2,3/3.5.4.6*	*1,2-Idd18.2/3-Idd27*			1*(Ampd1)* 87*(Ampd3)*	7 *(Ampd3)*
Purine nucleoside phosphorylase	*Pnp/2.4.2.1*		1			
Xanthine dehydrogenase/oxidase	*Xdh/1.17.1.4/1.17.3.2*		3	2 (1/1)	66	
Adenosine deaminase	*Ada/3.5.4.4*				3	
Adenosine kinase	*Adk/2.7.1.20*		3*		339	
ecto-5′-nucleotidase/CD73	*Nt5e/3.1.3.5*	*Idd 2*			59	
Deoxycytidine kinase	*Dck/2.7.1.74*					
Ectonucleoside triphosphate diphosphohydrolase 1/CD39	*Entpd1/3.6.1.5*				2	
Ectonucleoside triphosphate diphosphohydrolase (adenosinetriphosphatase)	*Entpd2/3.6.1.3*			2 (0/2)	3	1
Nucleoside-diphosphate kinase	*Nme7/2.7.4.6*		1			4
Isocitrate dehydrogenase	*ldh3a,3b,3g/1.1.1.41*	*Idd2/Idd13*			2	
Succinate-Coenzyme A ligase	*Suclg1/Suclg2/Sucla2 6.2.1.4*				79	2
Succinate dehydrogenase complex	*Sdha,b,c,d/1.3.5.1*	*a-Idd14/d-Idd2*	3 (Sdha)1(Sdhb) 3(Sdhc)	2 (0/2)	15*(Sdha)* 50*(Sdhb)* 52*(Sdhc)*	3*
Fumarate hydratase 1	*Fh1/4.2.1.2*				3	
Malate dehydrogenase 1	*Mdh1,2/1.1.1.37*				19	
3-hydroxy-3-mehtylglutaryl-CoenzymeA reductase	*Hmgcr/1.1.1.34*	*Idd14*				
Pannexin (Panx 3- imp in cartilage)	*Panx1,2,3*	*Panx3-Idd27*			12	
P2X purinoreceptor	*P2rx1,2,3,4,5,6,7*		20	1 (0/1)	68	
P2Y purinergic receptor	*P2ry1,2,4,6,10,12,13,14*	*P2ry2,6-Idd27*	8		19*(P2ry2)* 10*(P2ry6)* 7*(P2ry12)*	

* Defined locus in regulatory region.

Out of the 32 enzymes, five enzymes were found to be present in previously defined *Idd* regions (Table 1). Eight non synonymous exon SNPs (one in L-amino acid oxidase1, one in ureidopropionase, two in xanthine dehydrogenase, two in succinate dehydrogenase, two in ectonucleoside triphosphate diphosphohydrolase) resulted in amino acid substitutions, although only one SNPs (Xdh-rs29522348) was predicted by SIFT to be damaging utilizing both orthologue and homologue alignment. Of the 43 UTR SNPs in the enzymes, six SNPs (Adk-rs30494865, rs47796269, rs30728355, Sdhc-rs31555970, rs31555968, rs30909739) were found to be in the regulatory regions based on the dbSNP annotation.

In addition, the differences in the nucleic acid related molecules prompted us to investigate potential cell surface expressed receptors for these molecules. Three purinergic receptors, i.e. pannexin, P2X purinoreceptor and P2Y purinergic receptor were analyzed whereof two of the three receptors were found to be present in *Idd* regions (Table 1). When analyzed for the presence of SNPs, we found 28 located in UTR regions, one in exons (P2rx-rs13467733) and 116 SNPs in the intron regions.

## Discussion

It has previously been demonstrated that children with genetic predisposition for T1D development and that progressed to T1D display an altered serum metabolic profile compared to the group of non-progressors [3]. In this study we employed the NOD mouse with the hypothesis that this strain would display similar metabolic alteration as the T1D progressors, with the purpose that this model could give insight in the underlying molecular cause of these alterations. We found that NOD mice display a substantial altered metabolism compared to B6 already as a newborn and this difference was maintained throughout the 15 weeks analyzed.

In general the amino acid levels were slightly decreased in NOD prior to weaning, where after there was a general shift towards an increased level. It has been shown that the level of amino acids in the blood could be an indication of the status of glucose metabolism, i.e. higher levels of amino acids would indicate an increasing degree of catabolism [20]. Indeed the NOD mouse display higher levels of glucose already at birth and a reciprocal pattern of glucose levels to the amino acids was noted after weaning, supporting this notion. In addition, the level of isoleucine and valine were increased in NOD already in newborns (0 w) as well as right after weaning (3 w). The BCAAs have been shown to promote insulin secretion [21] which in turn could have an negative impact on the β-cells, i.e. excessive insulin secretion could lead to β-cell death [22].

Interestingly, in accordance with the Oresic study [3] we observed that glutamic acid was among the metabolites that discriminated the two strains the most. In addition to the increased level of glutamic acid a decreased level of glutamine was also observed as well. Both glutamine, and in particular glutamate, have effects on the immune system although the exact mechanism is still to be revealed [23]. Oresic et al hypothesized that initially anti-GAD antibodies may appear as a result of defective metabolism. In NOD mice anti-GAD antibodies appear between 3–4 weeks [24] in line with our observed increased level of glutamic acid. However, in contrast to the human study, no change of the glutamic acid levels in NOD relative to B6 was observed after this time point. In the analysis of metabolic pathways involving glutamic acid and related metabolites of the TCA cycle, i.e. alpha-ketoglutaric acid, we found that several enzymes, whereof some were located in previously defined *Idd* regions, displayed SNPs. There were a total of 1 078 SNPs in the introns of the analyzed enzymes which potentially could be critical for splicing either directly by altering donor, acceptor or branch sites. The efficiency of splicing could also be affected by intron SNPs due to splicing enhancer or silencer motifs alternatively by changing the secondary structure of the RNA. In addition, intron SNPs have been shown to affect enzyme activity [25], as well as a affect the expression levels of involved genes [26], [27]. A few non-synonomous exon SNPs were also found and the effect of these remains to be investigated. In general, the *in silico* genetic polymorphism analysis showed that several of the genes that are involved in metabolism of compounds highlighted both in this and in the Oresic study, are present in *Idd* regions. Although this does not prove involvement or the exact mechanism of involvement, it, however, supports a possible link between the observed metabolic changes and the potential responsible gene. This provides an interesting area for further studies in order to understand the mechanism behind the observed metabolic differences and their relation to development of T1D. Indeed, more detailed functional studies of these enzymes are desired.

One of the most striking differences that we observed was the difference in the nucleic acid related molecules. Recently it was found that NOD mice express increased levels of ADA promoting autoreactive T cell activation and diabetes development [28]. We have found that enzymes involved in ATP and adenosine metabolism have both non synonymous as well as intron SNPs. This can lead to potentially faulty metabolism of ATP and adenosine. In this study, increased levels of inosine, a direct metabolite of adenosine degradation was found. This can be the result of increased expression of ADA or a defect in ADA and ecto-nucleotidase enzymes. In addition, non-synonymous SNPs (rs28232063, rs28232059) were found in the ecto-nucleosidase, Entpd2, which is responsible for breakdown of ATP. A defect in this enzyme could lead to increased amounts of available ATP, an immune activator, causing increased cell activation and possibly autoreactive T cell activation. When we examined potential receptors we found three that also displayed some polymorphism comparing NOD and B6. The combination of both ligand and receptor alterations that in themselves may not seem that substantial but in combination could promote disease is plausible. Indeed, the effect of the P2X_7_ purinergic receptor has recently been described to act in combination with CD38 in promotion of T1D in the NOD mouse [29].

Another interesting observation was that methyl-inositol was markedly increased in NOD throughout the study. In the BB rat increased amounts of a methyl-inositol, identified as ribitol, was also singled out as one of the most discriminative for development markers for onset of diabetes [5]. This compound is clearly of exogenous origin and thus this demonstrate that there were not only metabolic differences but also differences in ability to ingest and metabolize exogenous compounds between animals that develop T1D and those that do not. Also campesterol, which is a plant sterol, was taken up differently between the stains with NOD displaying lower levels than B6. This also resembles our previous observation in the BB rat, where sitosterol and other sterol compounds were found decreased before diabetes onset [5].

The possibility to predict T1D progressors at the pre-diabetic stage is of importance as prevention, compared to reversion, of T1D has proven much more efficient, in particular in the NOD mouse where a large number of interventions has been described, as reviewed in [30]. If and how the pathways identified in this study contribute to the development of T1D cannot be proven conclusively based on our data. The results do, however, indicate that NOD mice display similar metabolic perturbations to human T1D progressors. Thus, the combination of metabolic profiling studies including definition of the underlying molecular mechanisms, and intervention studies such as feeding of certain metabolites, alternatively pharmaceutical inhibition of certain enzymes, could be tested in NOD mice. The metabolic consequences as well as effects on T1D development could hence be linked and contribute to our understanding of the pathogenesis leading to T1D development.

## Supporting Information

Text S1 Detailed protocol.
(DOC)

Figure S1 **Experimental design for metabolite identification.**
(DOC)

Figure S2 **The measured values of the compounds found most significantly changed between B6 and NOD mice at time points 0, 3, 4, 5 and 15 weeks as accounted for in** **Figure 3** **.**
(DOC)

Table S1 Fold change of metabolites.
(DOC)
