# Supplementary material for: Altered Metabolic Signature in Pre-Diabetic NOD Mice
Source: PLoS One. 2012 Apr 13;7(4):e35445. doi: 10.1371/journal.pone.0035445 (PMC3326011; doi:10.1371/journal.pone.0035445)
Supplement: Text S1 — Detailed protocol. (DOC) [file pone.0035445.s001.doc]

# Text S1. Detailed protocol

# Metabolite extraction

Extraction of metabolites from plasma was undertaken following the protocol of A *et al*. [1], using an extraction mixture of methanol : water (9:1), containing 11 internal standard compounds. The stable isotope reference compounds, [^2^H_4_]-succinic acid, [^13^C_5_,^15^N]-glutamic acid, [^2^H_7_]-cholesterol, [1,2,3-^13^C_3_]-myristic acid, [^13^C_5_]-proline, and [^13^C_4_]-disodium 2-oxoglutarate were purchased from Cambridge Isotope Laboratories (Andover, MA), [^13^C_6_]- glucose, [^13^C_12_]-sucrose,[^13^C_4_]-hexadecanoic acid, and [^2^H_4_]-1,4-butane-diamine-2HCl, were from Campro (Veenendaal,The Netherlands), and 2-hydroxy-[^2^H_6_]-benzoic acid was from Icon (Summit, NJ).

# GC-MS analysis

An aliquot, 25 µL, of thawed plasma was added to Sarstedt safety cap tubes and 225 µL of the extraction mixture consisting of methanol:water (9:1) containing 11 isotopically labeled internal standards were added. The mixture was shaken for 2 min at 30 Hz and stored in an ice-bath for 2 hours before centrifugation in an Eppendorf centrifuge (Model 5417C) for 10 min at 4ºC and14000 rpm. 200 µL of the supernatant were transferred to GC-MS-vials and evaporated to dryness, using a Speedvac. The samples were then derivatized by shaking them for 10 min with 30 µL pyridine containing methoxyamine, 15 µg/µL, followed by incubation at 70ºC for 60 min. Following incubation at RT for 16 hours N-Methyl-N-trifluoroacetamide (MSTFA), 30 µL, containing 1 % Trimethylchlorosilane (TMCS) was added. The mixture was vortex-mixed and allowed to react for 1 hour before addition of 30 µL of heptane containing 15 ng/µL methyl stearate. The extracted and derivatized samples were placed in a Agilent 7683 Series auto sampler (Agilant, Atlanta, GA) and 1 µL was injected splitless into a Agilent 6980 GC equipped with a 10 m x 0.18 mm i.d. fused-silica capillary column chemically bonded with 0.18 Tm DB5-MS stationary phase (J&W Scientific, Folsom, CA) coupled to a Pegasus III TOFMS (Leco Corp., St Joseph, MI) mass spectrometer. The injector temperature was 270ºC, the septum purge flow rate was 20 ml min^-1^ and the purge was turned on after 60 s. The gas flow rate through the column was 1 ml min^-1^, the column temperature was held at 70ºC for 2 minutes, then increased by 40ºC min^-1^ to 320ºC, and held there for 2 min. Ions were generated by a 70 eV electron beam at an ionization current of 2.0 mA, and 30 mass spectra s^-1^ were recorded in the mass range from m/z 50 to 800, after a solvent delay of 170 s. The ion source was maintained at 200ºC.

# Data Processing GC-MS

All non-processed MS-files from the metabolic analysis were exported from the ChromaTOF software in NetCDF format to MATLAB™ software 2006b (Mathworks, Natick, MA, USA), in which all data pre-treatment procedures, such as base-line correction chromatogram alignment, data compression and Hierarchical Multivariate Curve Resolution (H-MCR) were performed using custom scripts as described by Jonsson *et al.* [2]. All manual integrations were performed using ChromaTOF 2.12 software (Leco Corp., St Joseph, MI, USA) or in-house MATLAB scripts. The data processing protocols resulted in peak areas for the derivatized metabolites and corresponding mass spectra.

# Metabolite libraries and metabolite identification

The metabolites were identified by comparison of retention indices and mass spectra with data in commercial, as well as in-house, retention indexes and mass spectra libraries using NIST MS Search 2.0 (National Institute of Standards and Technology, 2001).

# Quantification of identified metabolites

The data processing of the GC-MS data using the H-MCR script resulted in initial datasets. All variables were checked manually and variables originating from internal standards and processing artifacts excluded. Additionally, chromatographic peaks originating from one compound but split during the data processing were re-processed using the H-MCR program or by manual integration. For study 1 GC-MS and UPLC-MS datasets were combined before modeling and doublet metabolites were removed. In all, semi quantitative data was achieved for 267 (study 1) / 240 (Validation study) putative metabolites.

# Sample normalization GC-TOF-MS

The dataset was normalized with the aid of the 11 added internal standards; a non-centered principal component analysis (PCA) model was built on the basis of the intensity of selected ions originating from the internal standard compounds. The magnitude of the PCA model t1-score of a given sample was taken as a general measure of the intensity [3]. Differences in intensity between samples are primarily expected to originate from differences in the system inlet efficiency. These effects are removed by dividing all measured metabolite intensities with the PCA model score t1-value for the corresponding sample.

# References

1. A, J., et al., *Extraction and GC/MS Analysis of the Human Blood Plasma Metabolome.* Anal. Chem., 2005. **77**(24): p. 8086-8094.

2. Jonsson, P., et al., *High-Throughput Data Analysis for Detecting and Identifying Differences between Samples in GC/MS-Based Metabolomic Analyses.* Anal. Chem., 2005. **77**(17): p. 5635-5642.

# Further reading of interest:

1. Redestig, H., et al., *Compensation for Systematic Cross-Contribution Improves Normalization of Mass Spectrometry Based Metabolomics Data.* Analytical Chemistry, 2009. **81**(19): p. 7974-7980.

2. Bolstad, B.M., et al., *A comparison of normalization methods for high density oligonucleotide array data based on variance and bias.* Bioinformatics, 2003. **19**(2): p. 185-193.

3. Trygg, J. and S. Wold, *Orthogonal projections to latent structures (O-PLS).* Journal of Chemometrics, 2002. **16**(3): p. 119-128.

4. Eriksson, L.J., E.; Kettaneh-Wold, N.; Wold, S., *Multi- and Megavariate Data Analysis - Principles and Applications*. 1 ed. Umetrics Academy. 2001, Umeå: Umetrics Academy. 531.

5. Bylesjö, M., et al., *OPLS discriminant analysis: combining the strengths of PLS-DA and SIMCA classification.* Journal of Chemometrics, 2006. **20**(8-10): p. 341-351.

6. Madsen, R., et al. http://dx.doi.org/10.1016/j.aca.2009.11.042
