# Supplementary figures and images for: Altered Metabolic Signature in Pre-Diabetic NOD Mice
Source: PLoS One. 2012 Apr 13;7(4):e35445. doi: 10.1371/journal.pone.0035445 (PMC3326011; doi:10.1371/journal.pone.0035445)

**Figure S1**


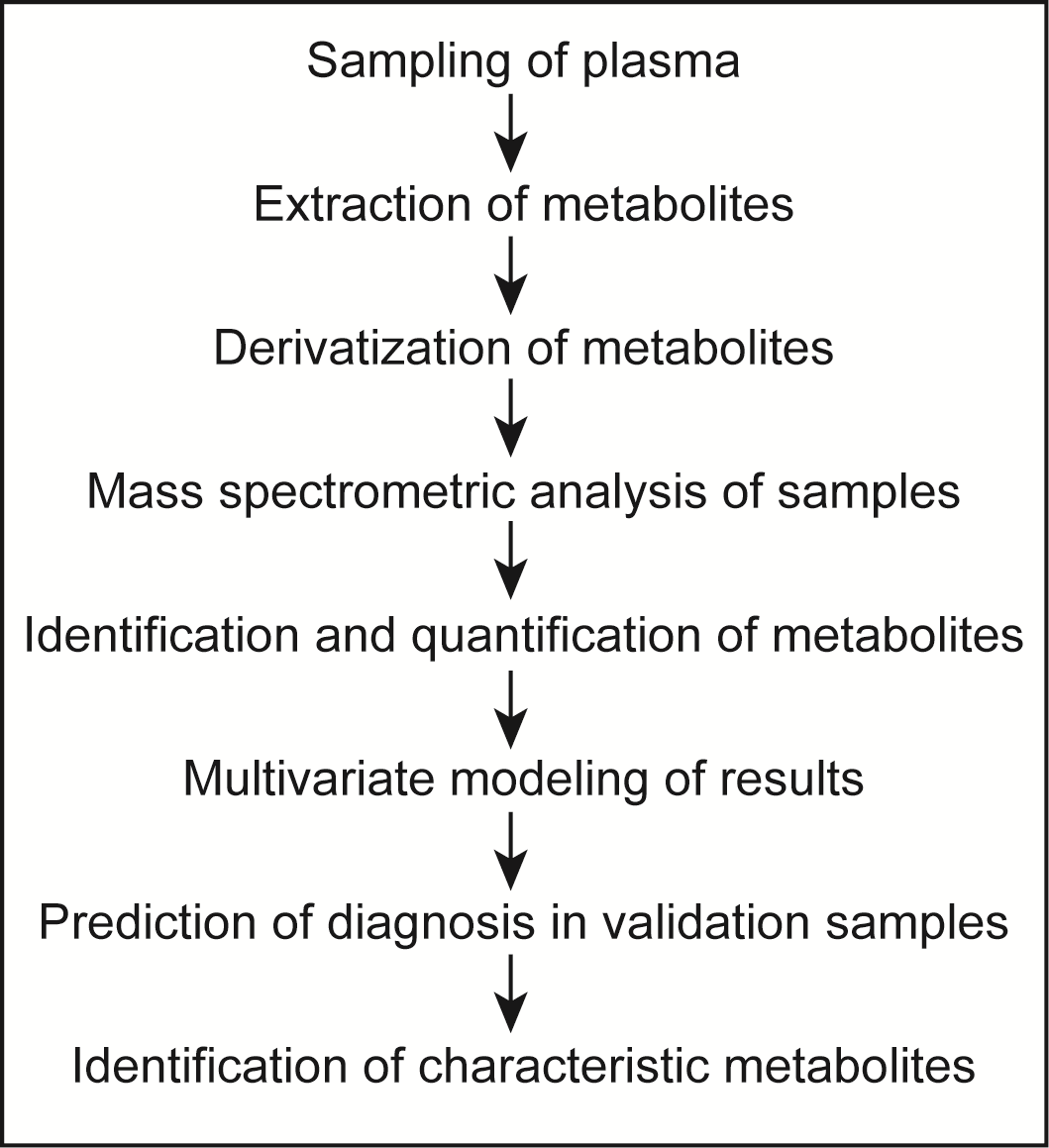


Figure S1. Experimental design for metabolite identification.

Supplement: Figure S1 — Experimental design for metabolite identification. (DOC) [file pone.0035445.s002.doc]
