# Supplementary material for: Altered Metabolic Signature in Pre-Diabetic NOD Mice
Source: PLoS One. 2012 Apr 13;7(4):e35445. doi: 10.1371/journal.pone.0035445 (PMC3326011; doi:10.1371/journal.pone.0035445)
Supplement: Figure S2 — The measured values of the compounds found most significantly changed between B6 and NOD mice at time points 0, 3, 4, 5 and 15 weeks as accounted for in Figure 3 . (DOC) [file pone.0035445.s003.doc]

**Figure S2**

**
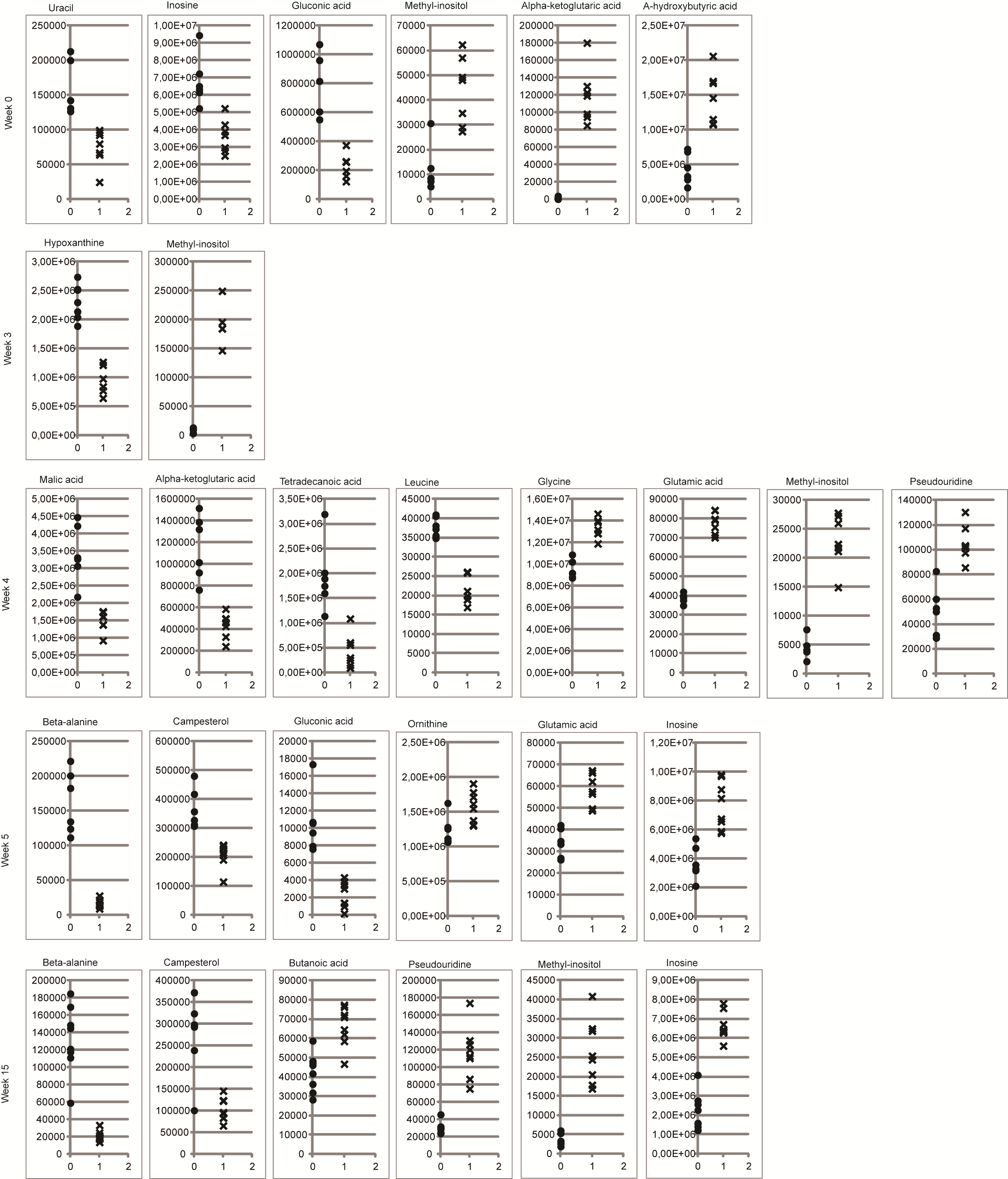
**

Figure S2. The measured values of the compounds found most significantly changed between B6 and NOD mice at time points 0, 3, 4, 5 and 15 weeks as accounted for in Figure 3.
