# Supplementary material for: Altered Metabolic Signature in Pre-Diabetic NOD Mice
Source: PLoS One. 2012 Apr 13;7(4):e35445. doi: 10.1371/journal.pone.0035445 (PMC3326011; doi:10.1371/journal.pone.0035445)
Supplement: Table S1 — Fold change of metabolites. (DOC) [file pone.0035445.s004.doc]

**Table S1. Fold change of metabolites.**

|  | **Week 2-3** | | **Week 3-4** | |
| --- | --- | --- | --- | --- |
|  | **Fold change NOD** | **Fold change**  **B6** | **Fold change NOD** | **Fold change**  **B6** |
| **Succinic acid** | -1,42 | -1,41 | 1,63 | 1,04 |
| **Citric acid** | 1,05 | 1,28 | 1,16 | 1,01 |
| **Fumaric acid** | 1,47 | 1,40 | -1,18 | 1,48 |
| **Malic acid** | -1,29 | 2,91 | 2,03 | 1,38 |
| **Alpha-ketoglutaric acid** | 1,76 | 8,16 | 1,81 | 2,86 |
| **Butanoic acid** | -1,78 | -1,89 | -1,47 | -1,35 |
| **Taurine** | -1,40 | -1,68 | -1,18 | -1,02 |
| **Threonic acid** | -1,21 | -1,48 | -1,14 | 1,10 |
| **Glycolic acid** | 1,60 | 8,35 | 2,75 | -2,15 |
| **Dihydroxy-butanoic acid** | -1,50 | -2,04 | -1,07 | 1,03 |
| **Alpha-hydroxy-butyric acid** | -1,20 | -5,15 | -5,95 | 1,28 |
| **Gluconic acid** | 7,19 | -3,95 | 2,24 | 1,02 |
| **Uracil** | -1,49 | -1,04 | -1,46 | -2,29 |
| **Guanine** | 1,10 | 2,06 | 1,60 | -1,50 |
| **Hypoxanthine** | -1,46 | 1,67 | -1,62 | -3,75 |
| **Uridine** | -3,29 | -5,92 | 1,07 | 1,47 |
| **Inosine** | 1,09 | -1,05 | -1,86 | 1,39 |
| **Guanosine** | -2,14 | -16,21 | -5,95 | 7,30 |
| **Pseudouridine** | -1,69 | -2,67 | -1,43 | 1,10 |
| **Glycerol-3-phosphate** | 1,19 | -1,55 | -1,13 | 1,02 |
| **Glycerol-2-phosphate** | 3,54 | -1,15 | -3,12 | 1,29 |
| **Lactic acid** | 2,12 | 1,86 | 1,24 | -1,46 |
| **Lactose** | 2,02 | 1,30 | 1,15 | 1,53 |
| **Maltose** | -4,27 | -6,67 | -4,90 | -1,17 |
| **Myo-inositol** | -1,52 | -1,55 | -1,18 | -1,16 |
| **Melezitose** | 1,66 | 14,20 | 9,53 | 2,06 |
| **Glucose-6-phosphate** | -2,58 | -3,86 | 1,46 | -1,09 |
| **Methyl-inositol** | 2,28 | -2,14 | -8,26 | -2,08 |
| **Galactono-1,4-lactone** | -1,44 | -1,68 | 1,30 | 2,78 |
| **Glucose** | -1,19 | -1,58 | 1,15 | 1,90 |
| **Methylglucopyranoside** | -1,08 | -2,33 | -1,05 | 2,99 |
| **Nonamide** | -2,15 | 1,56 | 4,30 | 3,19 |
| **Glycerol** | 1,21 | 1,00 | -1,47 | 1,22 |
| **Campesterol** | -3,98 | -2,01 | 8,18 | 3,97 |
| **Cholesterol** | -1,32 | -2,00 | -1,70 | 1,41 |
| **Decanoic acid** | -2,42 | -9,26 | -3,19 | -1,49 |
| **Heptanoic acid** | 1,16 | -1,03 | 1,16 | 1,20 |
| **Nonanoic acid** | -1,08 | -1,06 | 1,16 | 1,02 |
| **Stearic acid** | 1,07 | 1,07 | 1,01 | 1,27 |
| **Arachidonic acid** | 1,24 | -1,43 | -1,62 | -1,09 |
| **Oleic acid** | -1,42 | 1,31 | 2,70 | 1,92 |
| **Hexadecanoic acid** | -1,11 | -1,04 | 2,00 | 2,33 |
| **Elaidic acid** | -1,48 | 1,16 | 2,64 | 1,92 |
| **Linoleic acid** | -1,02 | 1,29 | 1,58 | 1,19 |
| **Docosahexanoic acid** | 1,02 | -1,08 | -1,56 | 1,29 |
| **n-Hexadecanoic acid** | -1,41 | -2,26 | 1,39 | 4,81 |
| **Lauric acid** | -2,57 | -6,02 | -15,67 | -3,86 |
| **Tetradecanoic acid** | -1,85 | -2,15 | -3,42 | -1,04 |
| **Palmitoleic acid** | -1,17 | 1,80 | 4,04 | 5,59 |
| **2,5-diaminovalerolactam** | -1,34 | -1,29 | 1,13 | -1,25 |
| **Pantothenic acid** | -1,37 | -3,22 | -6,62 | -2,09 |
| **Alpha-tocopherol** | 1,24 | 1,23 | 1,30 | 1,27 |
| **Allantoin** | -1,06 | -1,10 | 1,32 | 2,04 |
| **Phosphoric acid** | -1,45 | -1,66 | 1,08 | 1,05 |
| **Ornithine** | -1,36 | -1,21 | 1,06 | -1,57 |
| **Putrescine** | -1,37 | -1,71 | -1,18 | 1,02 |
| **Tyramine** | -1,53 | -1,38 | 1,40 | 1,03 |
| **Aminomalonic acid** | 1,12 | 1,30 | 1,22 | -1,30 |
| **Cystine** | -1,45 | -3,53 | -3,86 | 1,01 |
| **Ethanolamine** | 1,22 | 1,58 | 1,17 | -1,18 |
| **Urea** | -1,24 | -1,65 | 1,55 | 1,60 |
| **Allothreonine** | -1,43 | -1,43 | 1,50 | -1,14 |
| **Homoserine** | -1,42 | -1,37 | 1,46 | -1,13 |
| **Pyroglutamic acid** | -1,48 | -1,36 | 1,30 | -1,03 |
| **Creatinine** | -2,53 | -2,54 | 1,02 | -1,07 |
| **4-hydroxy proline** | -1,90 | -1,40 | 1,47 | -1,27 |
| **Hippuric acid** | 1,19 | 1,54 | 1,12 | 1,01 |
| **Cadaverine** | 1,29 | 1,81 | 1,13 | 1,03 |
| **5-hydroxy-tryptamine** | 1,40 | 2,36 | 1,90 | -1,20 |
| **Beta-alanine** | -2,05 | -1,15 | 1,12 | 2,47 |
| **Isoleucine** | -1,35 | -1,39 | 1,75 | 1,12 |
| **Valine** | -1,38 | -1,39 | 1,53 | 1,03 |
| **Cysteine** | 1,06 | 1,40 | 1,43 | -1,45 |
| **Methionine** | -1,80 | -1,69 | 1,20 | -1,03 |
| **Alanine** | 1,07 | 1,85 | 1,39 | -1,50 |
| **Tryptophan** | 1,08 | -1,17 | 1,11 | 1,06 |
| **Phenylalanine** | -1,23 | -1,22 | 1,40 | -1,23 |
| **Threonine** | -1,42 | -1,39 | 1,50 | -1,13 |
| **Lysine** | -1,72 | -1,43 | 1,53 | -1,02 |
| **Tyrosine** | -1,97 | -1,77 | 1,09 | -1,16 |
| **Glycine** | 1,16 | 1,80 | 1,01 | -2,34 |
| **Arginine** | -1,22 | 1,03 | 1,31 | -1,41 |
| **Aspartate** | -1,46 | -1,39 | 1,51 | -1,15 |
| **Serine** | -1,33 | -1,24 | -1,01 | -1,67 |
| **Glutamic acid** | -2,56 | -2,34 | 1,08 | -2,05 |
| **Proline** | -1,79 | -1,30 | 1,41 | -3,13 |
| **Asparagine** | -1,67 | -1,37 | 1,38 | -1,11 |
| **Glutamine** | -1,32 | 1,20 | 1,70 | -1,17 |
| **Leucine** | 1,22 | 1,11 | -1,54 | 1,27 |
